# Therapeutic Management of Feline Chronic Gingivostomatitis: A Systematic Review of the Literature

**DOI:** 10.3389/fvets.2016.00054

**Published:** 2016-07-18

**Authors:** Jenna N. Winer, Boaz Arzi, Frank J. M. Verstraete

**Affiliations:** ^1^Dentistry and Oral Surgery Service, William R. Pritchard Veterinary Medical Teaching Hospital, School of Veterinary Medicine, University of California Davis, Davis, CA, USA; ^2^Department of Surgical and Radiological Sciences, School of Veterinary Medicine, University of California Davis, Davis, CA, USA

**Keywords:** feline chronic gingivostomatitis, stomatitis, therapy, management, systematic review

## Abstract

Feline chronic gingivostomatitis (FCGS) is a disease characterized by protracted and potentially debilitating oral inflammation in cats, the etiology of which is currently unknown. The purpose of this review is to apply an evidence-based medicine approach to systematically review and critically evaluate the scientific literature reporting the outcome of medical and surgical management of FCGS. Those articles meeting inclusion criteria were reviewed and assigned an “Experimental Design Grade” (EDG) and an “Evidence Grade” (EG) in order to score relative strength of study design and produced data. Studies were evaluated and compared, especially highlighting the treatments, the outcomes, and the therapeutic success rates. This review found a lack of consistency between articles’ data, rendering direct comparison of results unreliable. The field of FCGS research, and ultimately patient care, would benefit from standardizing studies by adopting use of a consistent semi-quantitative scoring system and extending follow-up duration. Future researchers should commit to large prospective studies that compare existing treatments and demonstrate the promise of new treatments.

## Introduction

Feline chronic gingivostomatitis (FCGS) is a painful, often debilitating, condition in cats characterized by protracted oral inflammation typically lasting months to years. More specifically, it is differentiated from gingivitis when the inflammation crosses the mucogingival junction and extends to the buccal and caudal oral mucosa; classically, there are erosive and/or proliferative inflammatory mucosal lesions lateral to the palatoglossal folds (1, 2). The reported prevalence of FCGS ranges from 0.7 to 12.0% (3–6). Given that there are ~74–96 million cats owned in the United States (7), this translates to an estimated disease burden of at least 500,000 and upwards of 11 million cats.

Histologically, lesions are primarily infiltrated by lymphocytes and plasma cells, with fewer neutrophils, macrophage-like cells, and mast cells (8, 9). In addition, it has recently been noted that CD3+ T cells are present within the epithelium and submucosa of oral mucosa effaced by FCGS, whereas CD20+ B cells are mainly present within the subepithelial stroma (10). Histology is useful to confirm the diagnosis, and is necessary for academic and research purposes. However, clinical appearance and clinical signs may be sufficient for diagnosis. Affected cats may suffer from moderate to severe oral pain, halitosis, ptyalism, decreased grooming, hyporexia, weight loss, irritability, withdrawn behavior, and/or decreased activity (11, 12). Quality of life can be so severely affected that owners elect for humane euthanasia (3, 10, 13).

The etiology of FCGS remains elusive, but it is generally accepted that FCGS arises from an inappropriate immune response to oral antigenic stimulation, potentially multifactorial in nature and possibly with varying inciting causes (12, 14, 15). Myriad maladies have been implicated, from systemic pathogens (feline calicivirus, herpesvirus, leukemia virus, immunodeficiency virus, and *Bartonella*), to dental disease (feline resorptive lesions, periodontal disease), to hypersensitivity (overreaction to plaque bacteria, food allergies) (12, 14, 16–27). Detection of circulating T cells in cats suffering from FCGS supports the theory that the disease arises from an aberrant response to chronic, oral, antigenic stimulation stemming from clinical or subclinical viral infections (10, 14, 18, 27, 28).

Just as the underlying cause of FCGS is yet to be determined, a satisfactorily consistent and successful treatment regime is yet to be discovered. Many therapies have been pursued the past few decades, generally categorized as either medical or surgical management. The mainstay of medical therapy has traditionally been immunosuppression [i.e., corticosteroids (28) or cyclosporine (12)], while surgical treatment involves the extraction of premolar and molar teeth or the full dentition. These treatments are far from benign, with possible adverse effects ranging from polyuria, polydipsia, secondary diabetes mellitus, skin fragility, diminishing effectiveness over time (medical management), to postoperative pain and reduced function, owner psychological distress, and financial expense (surgical management). In order to discover an efficacious therapy with minimal side effects and in order to test the effectiveness of dental extractions, multiple studies have been conducted assessing the outcome of various therapies.

It may be postulated that current treatment options have remained unrewarding, with variable response rates, because of the multifactorial nature of FCGS, or because the inciting cause may differ between patients. Until the exact etiology is discovered, and therapy can, thus, be targeted accordingly, it is important to critically evaluate current treatment alternatives by utilizing an evidence-based medicine approach. Evidence-based medicine encourages clinicians to make decisions powered by the best available evidence gained from the scientific method (29). In compiling and appraising the current literature on FCGS treatments, the benefits and limitations of therapies can be weighed, while ultimately highlighting the need for further high-quality research on this topic. The aim of this study was to apply an evidence-based medicine approach to systemically review and critically evaluate the scientific literature reporting the outcome of medical and surgical treatments for FCGS.

## Materials and Methods

A systematic literature search was performed, including articles available through February 6, 2016, for manuscripts relating to the treatment of FCGS. The on-line databases PubMed, Web of Science, and CAB Abstracts were searched using the following terms: [(cat OR cats OR feline OR felines) AND (stomatitis OR gingivostomatitis) AND (treatment)].

Inclusion and exclusion criteria for article selection were predetermined to reduce bias. Articles included in this study are those published in peer-reviewed journals that discuss spontaneously occurring FCGS and original data about its treatment in domestic cats. A clearly defined treatment protocol for each cat must be provided (i.e., consistent dose of medication given to each treatment cat). Articles were excluded if not written in the English language, if FCGS is presented as a sequela of another disease for which treatment is primarily aimed (such as feline leukemia virus or feline immunodeficiency virus), if treatment of FCGS from a different study is summarized or recapitulated without contribution of new experimental or at least anecdotal data, or if a follow-up period is not delineated.

The authors read the titles, abstracts, and/or full text of the publications yielded in the database searches to determine study eligibility. Those articles meeting inclusion criteria were reviewed and were awarded an “Experimental Design Grade” (EDG), a scoring system of strongest (I) to weakest (V) evidence modified from a grading system published by the Oxford Centre for Evidence-Based Medicine (Table 1) (30–33). Articles were also assigned an “Evidence Grade” (EG) of strongest (A) to weakest (C) evidence based on the type of outcome being measured, modified from a previously devised scoring system assessing quality of data (Table 2) (34). In reporting the success rate of the therapeutic intervention being tested, use of the term “cure” can be misleading given that the etiology of FCGS remains elusive; the phrases “resolution” of FCGS lesions and/or clinical signs or “clinical remission” are more appropriate and will, thus, be utilized in this review when describing clinical outcome.

**Table 1:** **Grading scheme used to score the quality of experimental design for manuscripts included in this systematic review, modified from a grading system published by the Oxford Centre for Evidence-Based Medicine (30)**.

Experimental design grade (EDG)	Categories of therapeutic FCGS studies
I	Randomized, controlled, double-blinded prospective clinical trials
II	Prospective clinical trials (± control group)
III	Retrospective case series, *n* > 10; case–control study
IV	Retrospective case series, *n* < 10
V	Single patient case report; expert opinion

**Table 2:** **Grading scheme used to score the quality of data for manuscripts included in this systematic review, modified from a previously devised scoring system (34)**.

Evidence grade^a^ (EG)	For FCGS treatment outcome being measured
A	Histology
B	Semi-quantitative scoring system of lesion gross appearance and/or owner-reported clinical signs
C	Subjective outcome measures of lesion gross appearance and/or owner-reported clinical signs without a defined semi-quantitative scoring system

*^a^If a combination of outcome types were reported, the higher evidence grade was assigned (A > B > C)*.

The following data were extracted from each article included in this study: the name of the first (or only) author, publication year, the number of cats receiving treatment, the number of cats serving as controls, treatment(s) being tested, the control treatment or placebo (if applicable), the outcome being measured, duration of follow-up, the percentage of cats receiving treatment that significantly improved or went into clinical remission per the authors, the percentage of cats receiving the control medication or placebo that significantly improved or went into clinical remission per the authors, and the study’s funding source (Table 3). Table 3 was arranged by publication year, so that trends over time could be appreciated.

**Table 3:** **Summary of the data collected from the manuscripts included in this systematic review**.

Reference	Publication year	n_t_^a^	n_c_^b^	Treatment tested; control administered, if applicable	Outcome being measured	Duration of follow-up (months)	% n_t_^c^; % n_c_^d^	EDG	EG	Funding source
Kavanagh (43)	1998	1	0	Zincreo germicidal astringent obtundent (topically)	Clinical signs as reported per owner	0.5	100; n/a	V	C	None stated
Mayr et al. (38)	1991	33	39	Local paramunization with PIND-ORF (parapoxvirus ovis); “conventional treatment”	Not explicitly discussed (implied gross appearance of oral lesions)	6–18	42; 13	II	C	None stated
Hennet (39)	1997	30	0	Dental extractions: 24 cats had all premolar and molar teeth extracted, 2 had full-mouth extractions, 4 had one to five premolar teeth remaining	Semi-quantitative scoring system combining gross appearance of oral lesions, need for ongoing medical management, owner-reported clinical signs	11–24	80 (60); n/a	III	B	None stated
Addie et al. (44)	2003	1	0	Thalidomide + Lactoferrin powder (topically)	Calicivirus shedding; gross appearance of oral lesions	22	100; n/a	V	C	Morinaga Foods; Companion Animal Diagnostics, University of Glasgow
Baird (45)	2005	1	0	Extraction of all premolar and molar teeth	Gross appearance of oral lesions	2.2	100; n/a	V	C	None stated
Vercelli et al. (40)	2006	8	0	Cyclosporine	Semi-quantitative scoring system of gross appearance of oral lesions	3	100 (50); n/a	III	B	None stated
Southerden and Gorrel (46)	2006	1	0	Recombinant feline interferon omega	Gross appearance of oral lesions	6	100; n/a	V	C	None stated
Lewis et al. (47)	2007	1	0	Carbon dioxide laser	Gross appearance of oral lesions	36	100; n/a	V	C	None stated
Bellei et al. (11)	2008	21	0	Extraction of all premolar and molar teeth	Semi-quantitative scoring system of gross appearance of oral lesions	0.75	80.9 (57.1); n/a	II	B	None stated
Hennet et al. (28)	2011	19	11	Recombinant feline interferon omega (topically); tapering course of prednisolone	Semi-quantitative scoring system of gross appearance of oral lesions and a variety of other clinical signs	3	45 (10); 23 (7.7)	I	B	Virbac SAS, France
Corbee et al. (36)	2012	7	7	Diet with 10:1 ratio of omega-6 polyunsaturated fatty acid (PUFA) to omega-3 PUFA after premolar and molar teeth extractions; diet with 40:1 ratio of omega-6 PUFA to omega-3 PUFA	Semi-quantitative scoring system of gross appearance of oral lesions	1	Not explicitly stated by authors – Jenna N. Winer’s interpretation: 57; 85	I	B	None stated
Leal et al. (42)	2013	2	0	Recombinant feline interferon omega	Gross appearance of oral lesions and owner report of clinical signs	5–6	100; n/a	IV	C	None stated
Lommer (12)	2013	9	7	Cyclosporine; cod liver oil with tuna flavoring (placebo)	Semi-quantitative scoring system of gross appearance of oral lesions and a variety of other clinical signs	1.5	77.8 (45.5); 14.3	I	B	Academy of Veterinary Dentistry
Hung et al. (37)	2014	5^e^	5	Bovine lactoferrin oral spray + piroxicam; piroxicam	Histology; semi-quantitative scoring system of gross appearance of oral lesions, clinical signs, and quality of life	3	77; not explicitly stated	I	A	Happy Harvest Corporation
Jennings et al. (41)	2015	95	0	Dental extractions: full mouth or premolar + molar extractions	Semi-quantitative scoring system combining gross appearance of oral lesions, need for ongoing medical management, owner-reported clinical signs	1–88.5 (mean 7.7)	67.4 (28.4); n/a	III	B	None stated
Arzi et al. (10)	2016	7	0	Fresh, autologous, adipose-derived mesenchymal stem cell IV injections	Histology (*n* = 3); semi-quantitative scoring system combining gross appearance of oral lesions and owner-reported clinical signs	6–24	71.4 (42.8); n/a	II	A	NIH, WINN Feline Foundation, San Francisco Foundation

*^a^n_t_, number of cats with FCGS to which trial treatment medication was administered (does not include cats that dropped out of study)*.

*^b^n_c_, number of cats with FCGS to which control medication/placebo was administered (does not include cats that dropped out of study)*.

*^c^% n_t_, percentage of n_t_ that went into clinical remission and/or significantly improved per authors; number in parentheses represents the percentage of n_t_ patients that went into clinical remission per authors (excludes cats that significantly improved but did not achieve remission)*.

*^d^% n_c_, percentage of n_c_ that went into clinical remission and/or significantly improved per authors; number in parentheses represents the percentage of n_c_ patients that went into clinical remission per authors (excludes cats that significantly improved but did not achieve remission)*.

*^e^After 4 weeks, control cats were converted to treatment cats; so for weeks 5–12 of this study, there were 10 cats receiving bovine lactoferrin oral spray + piroxicam PO*.

## Results

**Figure 1:**
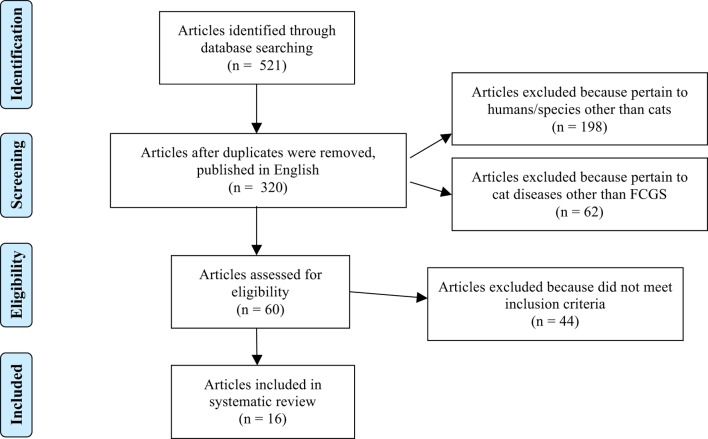
**Flowchart modified from the “Preferred Reporting Items for Systematic Reviews and Meta-Analysis (PRISMA)** (35) **guidelines,” demonstrating the process by which the search results were narrowed to the 16 articles included in this systematic review**.

Database searches yielded 521 articles for initial review; ultimately 16 met the inclusion criteria. A flowchart (Figure 1) modified from the “Preferred Reporting Items for Systematic Reviews and Meta-Analyses” (PRISMA) (35) guidelines is provided to outline the process by which the search results were narrowed to the 16 articles included in this systematic review. The most common reasons for study exclusion were manuscripts focusing on species other than cats (i.e., humans) as well as articles discussing treatment of other feline diseases, such as feline immunodeficiency virus, and mentioning stomatitis as a clinical sign or sequela. After assessing the included 16 studies, 4 were assigned an EDG of I (12, 28, 36, 37), 3 were assigned an EDG of II (10, 11, 38), 3 were assigned an EDG of III (39–41), 1 was assigned an EDG of IV (42), and 5 were assigned an EDG of V (43–47). Seven studies (43.8%) were prospective clinical trials, three studies were retrospective (18.7%), and six studies were case reports presenting the outcome of one or two cats (37.5%). There were 10 forms of medical management evaluated in these studies: Zincreo germicidal astringent obtundent (43), local paramunization with PIND-ORF (parapoxvirus ovis) (38), thalidomide (44), lactoferrin (37, 44), cyclosporine (12, 40), recombinant feline interferon omega (28, 42, 46), prednisolone (28), diet (36), piroxicam (37), and autologous mesenchymal stem cells (10). There were six studies that focused on surgical management, discussing efficacy of dental extractions (11, 36, 39, 41, 45) and use of carbon dioxide laser treatment as an adjunct to dental extractions (47). The reported mechanism of action of these treatments is as follows: local paramunization (38), thalidomide (44), lactoferrin (37, 44), cyclosporine (12, 40), recombinant feline interferon omega (28, 42, 46), and autologous mesenchymal stem cells (10) elicit immunomodulatory effects; prednisolone (28) and piroxicam (37) reduce inflammation; lactoferrin (37, 44) inhibits bacterial growth; recombinant feline interferon omega (28, 42, 46) impedes viral replication; diet (36) accelerates healing and reduces inflammation; carbon dioxide laser (47) removes proliferative tissue and stimulates fibrosis; and dental extractions reduce immune stimulation *via* eliminating plaque (11, 36, 39, 41, 45). The mechanism of action of Zincreo germicidal astringent obtundent was not directly reported (43).

The most common outcome measurement was utilization of semi-quantitative scoring systems ranking gross appearance of oral lesions and/or owner-reported clinical signs and/or the need for ongoing medical management (*n* = 7, 43.8%) (11, 12, 28, 36, 39–41), followed by reporting the qualitative gross appearance of oral lesions (*n* = 4, 25%) (42, 45–47). There were two studies (12.5%) that included both a semi-quantitative scoring system as well as mucosal histology post-treatment as their outcome measurements (10, 37), while another study measured shedding of calicivirus in addition to reporting the gross appearance of oral lesions (44). One study relied on clinical signs as reported by the owner over the phone (43). One study did not explicitly discuss the outcome measurement, which was inferred to be gross appearance of oral lesions (38). Thus, two studies produced level A evidence (10, 37), seven studies produced level B evidence (11, 12, 28, 36, 39–41), and seven studies produced level C evidence (38, 42–47).

### Surgical Management

There were six articles that focused on surgical management of FCGS (11, 36, 39, 41, 45, 47). Of these, two articles were graded as level V C case reports discussing single cats; one cat underwent premolar and molar tooth extractions and achieved clinical remission that was sustained for at least 2.2 months (45), while the other cat was treated adjunctively with a carbon dioxide laser after dental extractions and achieved clinical remission that was sustained for at least 36 months (47).

There were three articles specifically investigating the efficacy of dental extractions in groups of cats, each reporting grade B evidence (11, 39, 41). The earliest of the three articles (39) included a sample size of 30 cats, in which a 60% clinical remission rate was achieved (no visible lesions, no oral clinical signs), with an additional 20% of cats considered significantly improved without the need for ongoing therapy, 13.3% of cats showing little improvement, and 6.7% of cats making no improvement. The next article (11) assessed the results of dental extractions performed on 21 cats; of these, 57.1% achieved clinical remission, 23.8% improved, and 19.1% suffered relapses. The results of these two articles are strikingly similar, with ~80% of both groups demonstrating substantial improvement or resolution of FCGS lesions and the remaining 20.0% of cats benefiting minimally or not at all from surgical treatment. The most recent and comprehensive article assessing dental extractions was a retrospective case series, including 95 cats treated with either full-mouth extractions or premolar and molar tooth extractions (41). This study reports that significantly fewer cats (28.4%, *P* = 0.002) recovered completely compared to the previous two studies (11, 39). While a greater percentage (39.0%) of cats showed substantial improvement, the authors point out that this was not statistically significantly different from the first extraction article’s (39) finding of 20.0% (*P* = 0.077). At the last recheck examination, 26.3% of their patients showed little improvement and required ongoing medical management [again, not statically significantly different from the first extraction article’s (39) finding, *P* = 0.214]. In this study, 6.3% of cats remained refractory to extraction treatment [yet again, not statically significantly different from the first extraction article’s (39) finding, *P*-value not provided]. Approximately one-third of the 95 cats had been treated with premolar and molar tooth extractions while the remaining approximately two-thirds were treated with full-mouth extractions; there was no significant difference in response to treatment between these groups (*P* = 0.377). These authors also found that treatment with antimicrobials, anti-inflammatories, or analgesics prior to or at the time of extractions was not associated with a better outcome. Cats that were reported to have resolution of abnormal behaviors associated with FCGS at the time of their first postoperative recheck had odds of a positive outcome (clinical remission or substantial improvement) 7.2 times as great as in cats without resolution. Just over two-thirds of the cats that achieved clinical remission or substantially improved did require additional medical management for a finite period of time beyond the immediate postoperative period, whereas the first study (39) reported no need for medical management in the 80% of cats that went into clinical remission or significantly improved, and the second study (11) did not discuss if ongoing medical management was necessary.

In the sixth article discussing dental extractions (36), the primary focus was not on surgical management, but instead the intent was to investigate if diets with differing omega-3 to omega-6 polyunsaturated fatty acid ratios would affect inflammation and wound healing when fed to cats postoperatively after premolar and molar tooth extractions. Cats’ FCGS was scored utilizing a five-point semi-quantitative scale examining the degree of inflammation preoperatively and 4 weeks postoperatively. While there was no statistically significant difference between groups fed different diets (*P* = 0.366), overall, there was significant improvement in FCGS scores of both groups (*P* = 0.017, *P* = 0.042), presumably owing to the dental extractions. With their data pooled, the 14 cats’ FCGS scores improved on average by 52.1% (range 0–72.0%).

### Medical Management

The remaining 10 articles explore the efficacy of various medical management therapies (10, 12, 28, 37, 38, 40, 42–44, 46).

There are two studies that test the efficacy of oral cyclosporine; in the first (40), cyclosporine was administered to a group of eight cats that were not previously treated for FCGS with dental extractions, while in the second (12), cyclosporine was administered to nine edentulous cats. In the former study, 50.0% of cats achieved clinical remission, while the remaining cats showed 40.0–70.0% improvement in their semi-quantitative lesion scores during the 6-month follow-up period. In the latter study, 45.5% of cats achieved clinical remission, while 77.8% of cats showed a >40.0% improvement in their semi-quantitative stomatitis scores over the 6-week study period; by contrast, only 1 of 7 (14.3%) of cats in the placebo group showed a >40% score improvement.

A relatively similar success rate was achieved in a pilot study exploring the efficacy of fresh, autologous, adipose-derived mesenchymal stem cells intravenously injected into seven cats (10). Over the 6- to 24-month follow-up period, 42.8% of cats (*n* = 3) went into clinical remission, 28.6% (*n* = 2) demonstrated substantial improvement, and 28.6% (*n* = 2) did not respond. This article was awarded a level A EG because it not only utilized a semi-quantitative scoring system but also included histology from cats that achieved either clinical remission, substantial improvement, or that failed to respond. Histology results paralleled clinical gross examination of the lesions. While harvesting mesenchymal stem cells from the patient’s fat may preclude this treatment from easily being adopted widely at this juncture, the authors state that additional investigations using fresh, allogeneic cells are ongoing. Another unique aspect of this article is that the authors identified a potential biomarker to predict therapeutic outcome of stem cell treatment.

There were three articles, each testing a different therapy [Zincreo (43), thalidomide with lactoferrin (44), and recombinant feline interferon omega (46)], that were graded V C and each yielded a 100% remission rate in their single cat patient. In another article (42), graded IV C, recombinant feline interferon omega was administered to two cats, yielding a 100% remission rate.

The remaining three articles that discuss medical management included a control group (28, 37, 38). In one study (38), the success of treating with local paramunization using PIND-ORF (parapoxvirus ovis) versus “conventional treatment” (the authors did not define the specific control therapy) was compared. There were 33 cats treated with paramunization, resulting in 42.0% of them achieving clinical remission or substantially improving, while only 13.0% of the 39 cats receiving “conventional treatment” achieved the same positive results. Another study (37) tested the efficacy of bovine lactoferrin administered with piroxicam versus piroxicam alone as a control; 77.0% of cats receiving both medications achieved clinical remission or substantially improved after 12 weeks, whereas the authors did not state the success rate of the control group, as control cases were converted to treatment cases after the fourth week. Finally, a study (28) compared the effectiveness of recombinant feline interferon omega in 19 cats versus the control treatment of prednisolone in 11 cats; 45.0% of the treatment cats substantially improved (10.0% of which achieved clinical remission), while 23.0% of control cats substantially improved (7.0% of which achieved clinical remission). However, these differences between treatment and control group were not statistically significant.

## Discussion

To the authors’ knowledge, this is the first systematic review of the literature analytically evaluating the outcome of studies on therapeutics for FCGS. It is prudent to critically and systematically evaluate this literature, especially when studies have thus far failed to identify a consistent clinical resolution for this condition.

### Lack of Statistical Power

Of the 16 articles included in this review, 6 were small retrospective case series or single patient case reports (EDG IV or V). Lacking any statistical power, these articles provide weak evidence. That being said, it is heartening that, in general, more recent studies have a higher EDG and EG than earlier studies. Ideally, studies would build off of the results of one another, expanding promising level V experiments to a level II or I clinical trial. However, in compiling FCGS articles, this is rarely the case. Instead, the literature is at times circuitous, expending efforts in studies that stand to change the overall conversation minimally. For example, after a level III B article (39) was published discussing efficacy of dental extractions in 30 cats, 8 years later a level V C case report (45) was published discussing success of premolar and molar tooth extractions in a single cat, which does not advance collective knowledge about FCGS treatment. Similarly, a level V C single patient case report (46) published on recombinant feline interferon omega was followed 7 years later by a level IV C case report (42) published testing the same drug in two cats with an almost identical follow-up time and result.

### Inconsistent Outcome Measurements

This review identified that 9 of the 16 articles assessed outcome of FCGS treatment *via* semi-quantitative scoring systems. A novel custom scoring system was developed and defined in four of these articles (28, 36, 39, 40), while two articles utilized the same scoring system (10, 12), and three articles each modeled their scoring system off of different previously utilized scoring systems (11, 37, 41). This represents a significant obstacle both to the comparison of existing results and for the planning of future studies. Recorded outcome measures in articles included in this study include gross examination of oral lesions by veterinarians, other physical exam findings (such as body weight or body condition score or prominence of mandibular lymph nodes), owner-reported clinical signs in the home setting, and/or owner-perceived quality of life for their cat. This review not only found little consistency between articles in the scoring system used to record outcome but also in the duration of follow-up. Follow-up periods ranged from 2 (43) to 354 weeks (41). The lack of consistency between studies in data reporting makes direct comparison of results problematic. In order to draw sound comparisons, ideally all studies would adopt the same validated outcome measure, with histology of oral lesions being the gold standard. Universal adoption of a standardized semi-quantitative scoring system validated with histopathology results would be ideal. A longer follow-up time is obviously superior to a shorter follow-up period, but at some point practicality limitations outweigh the desire to continue collecting data. No study has explored the time cutoff after which response to either medical or surgical therapy remains static; until such a study is performed, it is reasonable that prospective studies continue collecting data at least 6 months beyond plateaued response to treatment.

### Refractory versus Naïve FCGS

In analyzing articles whose focus is medical management, it is important to bear in mind if the subjects are refractory to historically performed dental extractions, if they have failed to respond to previous medical management attempts, or if their FCGS is naïve to any previous therapeutic intervention. For example, it is noteworthy that the clinical remission rate was strikingly similar for cats involved in the two cyclosporine studies (12, 40) despite the fact that one group of cats had been resistant to previous medical management (40), whereas the other group had been refractory to full-mouth extractions (12).

### Experimental Design Grade and Evidence Grade

The grading systems utilized in this study provide a framework for analyzing experiment design and strength of the data reported. The grading process is not intended to criticize specific articles, and it is important to bear in mind that each article has its own strengths and weaknesses. The key components that comprise a strong FCGS study include the following: a large enough study population to be representative, a prospective experimental design, a quantitative or at least semi-quantitative scoring system for disease severity, and a long enough duration of follow-up to convince the reader that results will be sustained indefinitely. None of the articles included in this review meets all four of the above criteria.

There were four articles awarded a level I EDG, in part because they included either a control group (28, 36, 37) or a placebo group (12). While it is generally accepted that studies are strengthened by the inclusion of a control or placebo group, this practice is potentially problematic when applied to FCGS treatment clinical trials. One such problem is deciding upon the treatment administered to the control group. Each of the controlled studies included in this systematic review tests a different control treatment, namely a corticosteroid (28), a non-steroidal anti-inflammatory (37), or a diet (36). While selection of these control group treatments was intended to provide internal validation *via* direct comparison within studies, it renders comparison across studies difficult, if not meaningless. Control therapies have not been standardized; it could be informative to define a standardized control therapy against which to compare novel therapies. A second problem is the ethical dilemma that arises when cats enrolled in control or placebo groups experience prolonged suffering by not receiving superior, or any, treatment. One approach could, thus, be to eliminate placebo and control groups altogether. A benefit of this action is that more cats would be available to receive and test a treatment. Furthermore, spontaneous recovery or significant clinical improvement has never been reported in cats with refractory FCGS, which may devalue control or placebo groups (10). However, best practice in study design strives for the inclusion of control and placebo groups. The ethical predicament that accompanies use of control and placebo groups could be mitigated by allowing for analgesic administration to all cats throughout the study period. Additionally, if the treatment being tested is found to be promising, the cats in the control or placebo groups could be converted to cats receiving treatment, as occurred in the single placebo-controlled study discussed in this systematic review (i.e., a randomized controlled crossover study with one of the two treatments being a placebo) (12).

There were two articles awarded a level A EG for including histology of lesions post-treatment (10, 37). While FCGS is frequently diagnosed on the basis of clinical appearance and clinical signs, it is still beneficial to perform histopathology, ideally before and after treatment. Histopathology is useful not only to confirm the diagnosis (i.e., differentiate it from other diseases, such as squamous cell carcinoma) but also to help further classify and understand the lesion. The current lack of knowledge regarding the etiology of FCGS and the extent of mucosal response to therapy can only definitively be assessed *via* histological and immunohistochemical means, which validate and compliment the subjective assessment of clinical appearance and clinical signs. The more histopathological knowledge we have of this disease, the more potential there is to discovering the underlying etiology(s) and the most efficacious treatments.

### Study Limitations

There are limitations that may potentially affect the results and conclusions produced by this study. Inclusion and exclusion criteria were pre-defined to minimize bias; however, these criteria resulted in the exclusion of potentially influential articles. For example, an article exploring the efficacy of bovine lactoferrin was excluded because the study lacked a standardized definition of FCGS, with one experimental cat’s disease described as “severe gingivitis with hemorrhage” (48). Another article reporting the outcome of various therapies, including corticosteroids, antibiotics, megestrol acetate, levamisole, azathioprine, cyclophosphamide, and injectable gold (aurothioglucose) was excluded due to lack of defined treatment protocol, with inconsistent drug doses and no reporting of which cats received which treatment(s) (16). Another article examining various medical therapies, including diet adjustment, megestrol acetate, antibiotics, corticosteroids, levamisole, and mouthwash, was excluded because of inconsistent treatment protocols as well as no defined follow-up timeframe (8).

A second potential limitation is that only treatments for which there have been peer-reviewed studies published in the English language could be included in this study. Although chlorambucil, vincristine, 5-fluorouracil, sulodexide, tacrolimus topical, colchicine, and lysine have all been suggested as possible therapies (23), to the authors’ knowledge, there are no published reports assessing their efficacy within the veterinary peer-reviewed literature.

Because of the heterogeneity in outcome measurements, study design, population sizes, and follow-up times, it was impossible to perform a meta-analysis of articles’ data, and, thus, results of each study have been reported individually.

### Future Directions

In considering treatment options for FCGS, veterinarians should strive to adopt an evidence-based approach to their therapeutic recommendations. While studies have shown the percentage of cats that respond to various medical and surgical interventions, clinicians must sift through these data and apply it to their individual patients. Many questions still remain – for which cats should medical management be attempted initially versus recommending surgical management at the time of initial diagnosis? At what point should medical management be discontinued in favor of switching to surgical management if a cat is failing to respond to therapy, versus administering an alternate medical management? Can we predict which cats will respond to various treatment types?

Veterinarians should strive to improve the quality of FCGS research and, thus, the evidence base available to inform colleagues’ therapeutic recommendations in order to optimize patient care. In addition, we should bear in mind both the strengths and weaknesses of the current literature when drawing conclusions. Large prospective studies are needed to compare existing treatments and demonstrate the promise of new treatments. With the emergence of novel and innovative therapeutics, the field of FCGS research would benefit from standardizing studies by adopting use of the same quantitative or semi-quantitative scoring system and extending follow-up duration to at least 6 months beyond plateaued response to treatment.

## Conclusion

The current peer-reviewed literature on FCGS therapeutic outcome has demonstrated the statistical success rates of various treatments, reporting the percentage of cats achieving clinical remission when treated with either medical or surgical management. Future studies recapitulating this same point of view may further refine the success rates of therapies already in use, but this may represent stagnation and not innovation. A subset of cats suffering from FCGS remains refractory to the treatments evaluated in this systematic review, and to the authors’ knowledge there is no therapy that has adequately and convincingly been proven to achieve a 100% clinical remission rate. This most likely harkens back to the nebulous underlying etiology of FCGS and our current lack of understanding of its pathogenesis (12, 14). The authors of this systematic review agree that full-mouth or near full-mouth dental extractions is the current standard of care for FCGS (10, 41), but can we do better than the recently reported complete remission rate of 28.4% (41) that surgical management attains? The “holy grail” of FCGS research is to discover the etiology of FCGS and to identify a treatment protocol that attains a near 100% remission rate. In order to progress toward this goal, a paradigm shift in FCGS research is necessary. New directions must be pursued, in which researchers focus on new and innovative treatment strategies, such as modulating cats’ immune responses underlying their oral inflammation. As this type of research is already underway (10), future additional studies are required in order to ultimately discover both the cause and the cure for FCGS.

## Author Contributions

JW performed literature searches, determined which articles met inclusion criteria, proposed use of Experimental Design Grade and Evidence Grade schemes, and primarily authored the manuscript. BA proposed the idea to write a review on feline chronic gingivostomatitis treatment outcomes, reviewed the articles identified *via* literature search and determined which met inclusion criteria, proposed idea to submit to Frontiers in Veterinary Science, and handled editing of manuscript. FV reviewed the articles identified *via* literature search and determined which met inclusion criteria, proposed use of the PRISMA flow diagram, and handled editing of manuscript.

## Conflict of Interest Statement

The authors declare that the research was conducted in the absence of any commercial or financial relationships that could be construed as a potential conflict of interest.
